# Fear appeals to promote better health behaviors: an investigation of potential mediators

**DOI:** 10.1080/21642850.2021.1947290

**Published:** 2021-07-06

**Authors:** Lisa Selma Moussaoui, Nancy Claxton, Olivier Desrichard

**Affiliations:** a Health Psychology Research Group, Psychology Section, Faculty of Psychology and Education Sciences, University of Geneva, Geneva, Switzerland; b Health Department, International Federation of Red Cross and Red Crescent Societies, Geneva, Switzerland

**Keywords:** Threatening communication, efficacy, defensive reactions, order effect, Extended parallel process model

## Abstract

**Background:** Fear appeals are widely used in health communication, despite conflicting views on their effectiveness. Unresolved issues include possible mediation mechanisms and the effect of defensive reactions aimed at controlling a perceived danger.

**Methods:** The present study compared the impact of three versions of an existing online course on how to prevent noncommunicable diseases. Participants, recruited in South America via a crowdsourcing platform, were divided randomly between three versions of the course – ‘threat only’/‘threat plus coping information’/‘coping information plus threat’ (reverse order). We then asked them to complete a questionnaire measuring perceived efficacy, perceived threat, defensive reactions, and intention to change unhealthy behaviors.

**Results:** Using a serial parallel mediation model to test the course's impact on our dependent variables did not reveal any significant differences between the three versions. Perceived efficacy was positively associated with intention to change behavior, as well as with lower suppression, lower reappraisal, and greater denial. Suppression was the only defensive reaction to be associated with intention to change behavior: greater suppression was linked to less intention to change.

**Conclusions:** Our results open interesting perspectives for research into defensive reactions.

## Introduction

Noncommunicable diseases (NCDs), which include cardiovascular diseases, cancer, chronic respiratory diseases, and diabetes, are the leading cause of death worldwide (WHO, 2018). Because tobacco use, physical inactivity, excess alcohol use, and unhealthy diet are risk factors for developing NCDs (WHO, 2002), promoting healthier lifestyles is an important way of preventing these diseases (Chokshi & Farley, 2014). Multiple behavior change interventions have been described as ‘the future of preventive medicine’ by Prochaska (2008, p. 281). Practical arguments in favor of targeting several behavior in one intervention are notably reduction of the costs and maximizing reach because of limited contact opportunities for health promotion (Prochaska, Spring, & Nigg, 2008). Theoretically, it is reasonable to expect that general theories, supposed to describe behavioral principles valid for several health behaviors, would work targeting multiple behaviors at a time (Noar, Chabot, & Zimmerman, 2008). Noar et al. (2008) mention that recipients of the intervention can learn behavior change principles that they can apply to several health behaviors. Empirical evidence is also available on clusters of behaviors (Sallis, Prochaska, & Taylor, 1999), and the changes in one behavior being mirrored in another behavior, for example, physical exercise and tobacco consumption (Nagaya, Yoshida, Takahashi, & Kawai, 2007; Unger, 1996; but see also Sarma et al., 2019 for inconclusive evidence).

One general principle frequently used in persuasive campaigns is highlighting the negative consequences of the behaviors (Higbee, 1969; Ruiter, Kessels, Peters, & Kok, 2014; Williams, 2012; Witte, 1996a), and has been applied independently to the four risk factors of NCDs (e.g. for diet: Bleakley et al., 2015; tobacco: Emery, Szczypka, Abril, Kim, & Vera, 2014; alcohol use: Fritzen & Mazer, 1975; physical activity: Redmond, Dong, & Frazier, 2015).

### Use of fear appeals in health communication

Fear appeals are widely used in health communications due to the intuitive belief that scaring people will make them change (Kok, Peters, Kessels, ten Hoor, & Ruiter, 2018), even though intuition is known to be an inadequate basis for building effective interventions (Wilson & Juarez, 2015). In fact, research into whether or not fear appeals are effective has failed to produce unequivocal findings, leading to an ongoing debate among scholars that dates back as least as far as Higbee (1969). Given this scholarly uncertainty, it is unsurprising that institutions also have varying opinions about the impact of fear appeals. For example, the Centers for Disease Control and Prevention (2014) favors using fear appeals whereas the Drug Free Action Alliance (2013) believes the technique to be ineffective. This debate was recently rekindled by Kok et al. (2018), whose conclusions elicited numerous comments (Borland, 2018; Brewer, Hall, & Noar, 2018; Malouff, 2018; Niederdeppe & Kemp, 2018; Peters & Shoots-Reinhard, 2018; Roberto, Mongeau, & Liu, 2018; White & Albarracín, 2018). On the one hand, opponents of using fear appeals in persuasive messages argue that they trigger defensive reactions (Kessels, Ruiter, & Jansma, 2010; van ‘t Riet & Ruiter, 2013) and that they are effective only in very rare cases (Kok et al., 2018). On the other hand, proponents argue that fear appeals positively affect attitudes, intentions, and behaviors, with one meta-analysis reporting an average effect size of *d* = 0.29 (Tannenbaum et al., 2015).

### Coping information

In contrast to the controversy over the effectiveness of fear appeals, there is general consensus – supported by three meta-analyses (Floyd, Prentice-Dunn, & Rogers, 2000; Milne, Sheeran, & Orbell, 2000; Witte & Allen, 2000) and recent individual studies (e.g. Ort & Fahr, 2018; Roberto, Mongeau, Liu, & Hashi, 2019) – that providing coping information, in other words, telling people how they can face a threat, is beneficial (Peters, Ruiter, ten Hoor, Kessels, & Kok, 2018). Several theoretical models have integrated coping information as a predictor of reactions to a threatening message. For example, the Extended Parallel Process Model (EPPM, Witte, 1992), which drew on the Drive Reduction Model (Hovland, Janis, & Kelley, 1953), Leventhal's Danger Control/Fear Control Framework (1970), Protection Motivation Theory (Rogers, 1975), and the Health Belief Model (Rosenstock, 1966), predicts that responses to a perceived threat will depend on the balance between perceived threat (severity and susceptibility) and perceived efficacy (self-efficacy and response efficacy). When perceived efficacy is high, individuals will be motivated to protect themselves and therefore accept the message as part of a danger control process. Conversely, when perceived efficacy is low, individuals will be motivated to defend themselves and therefore reject the message as part of a fear control process. Meta-analysis have shown that response efficacy is a stronger predictor of intention to change than the emotion of fear (Floyd et al., 2000; Witte & Allen, 2000). Response efficacy was notably studied by Lewis, Watson, and White (2010) as a mediator of the effect of emotional responses on message acceptance and message rejection. They showed that response efficacy was associated only with message rejection (i.e. defensive reactions) and not with message acceptance.

### Mediation mechanisms and defensive reactions

Although fear appeals have been intensively researched over the years, the subject continues to attract a great deal of attention. One issue that deserves more work is mediating process. Mongeau (2013) review the historical development of the field and discuss fear appeal explanations. Early work suggested that the mediating variable was the emotion of fear (Hovland et al., 1953), triggered by the threat and pushing the person toward resolving an unpleasant affective state. Later focus was more on the cognitive process, notably in the PMT (Rogers, 1975), in which the main variable is how much the person is motivated to protect themselves. More recent work by Witte (1992) an attempt to integrate both affective and cognitive process through the evaluation of perceived efficacy and perceived threat in a feedback loop with fear. Notwithstanding, Mongeau concludes that the state of the literature is similar to 1984, when Boster and himself claimed ‘none of the fear appeal explanations are consistent with the available evidence’ (Mongeau, 2013, p. 193).

The effect of defensive reactions is an unresolved issue. For example, contrary to their predictions, Owusu, So, and Popova (2019) found higher levels of negative emotions among participants who reported adaptative responses to cigarette warning labels than among participants who reported maladaptive responses. In addition, participants who reported maladaptive responses did not report higher levels of denial. Similarly, following a four-country study of reactance to cigarette labels, Cho et al. (2016) found that stronger reactance was associated with a slightly higher (rather than lower) likelihood a participant would try to stop smoking. An attempt to explain why fear might increase persuasion in some studies but heighten defensive reactions in others led Blondé and Girandola (2019) to suggest that not enough is known about the relationship between persuasion, information processing, and defensiveness. Indeed, few studies have simultaneously measured all three concepts. Participants in Blondé and Girandola's high fear condition had more negative attitudes toward the risky behavior and this effect was mediated by an increase in defensive reactions (operationalized as comparative optimism). Theoretical arguments (Blondé & Girandola, 2016; van ‘t Riet & Ruiter, 2013) also support the idea that defensive reactions might not necessarily lead to the reduction in danger control suggested by the EPPM.

### The present study

Despite the long history of research into the effects of fear appeals on persuasion and behavior change, disagreement still exists between the technique's opponents and proponents, and the role of defensive reactions remains unclear. The present study contributes to current research on this important health communication issue by examining possible mediators of the effects of fear appeals on improving health behaviors. To do this, we used ‘real-life’ material from the International Federation of Red Cross and Red Crescent Societies’ (IFRC) Healthy Lifestyle Community online course, which aims to prevent NCDs by raising awareness of risk factors and encouraging people to adopt healthier lifestyles. Like many tools in health promotion, the course focuses on the negative health effects of particular behaviors. Thus, it appeared a natural candidate for our study. We used the original version of the course (*threat-only*) as the control and compared it to a *threat-coping* version and a *coping-threat* (i.e. reverse order) version*.* We did this because the order in which coping information and threat are presented has been sometimes shown to have an effect, depending on characteristics such as the degree to which an individual already follows the advocated recommendations (Keller, 1999) or the level of distress the message elicits (Brown & West, 2015).

#### Hypotheses

**Figure 1.**
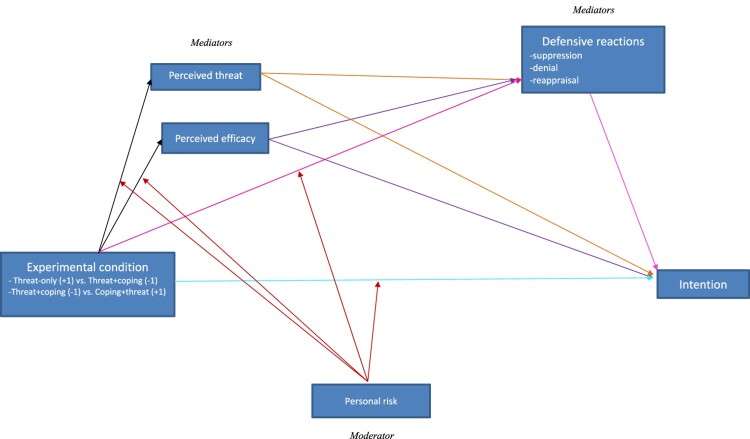
The postulated model.

The model we tested postulated a serial parallel mediation with personal risk of developing an NCD as a moderator (Figure 1).

The hypotheses we tested were as follows:

1. Experimental condition (X) will impact intention to change behavior (Y).
  1.1. Intention to change behavior will be weaker in the *threat-only* condition than in the *threat-coping* condition.
  1.2. In order to explore the difference between the *threat-coping* and *coping-threat* conditions we postulated that this difference will be moderated by personal risk of developing an NCD. Thus, we expected participants with a higher risk of developing an NCD to express greater intention to change behavior when they were exposed to the *coping-threat* version than when they were exposed to the *threat-coping* version. We expected to obtain the opposite result for participants with a lower risk of developing an NCD.
2. The effect of experimental condition (X) on intention to change behavior (Y) will be mediated by two parallel series of mediators. Thus the mediation effects of perceived efficacy and of perceived threat will themselves be mediated by defensive reactions (suppression, reappraisal, denial).
  2.1. Effect of experimental condition on efficacy and threat:
    2.1.1. Compared with the *threat-coping* condition, the *threat-only* condition will engender higher threat perception and lower efficacy.
    2.1.2. In order to explore the difference between the *threat-coping* and *coping-threat* conditions we postulated that their effects will be moderated by personal risk of developing an NCD. Hence, we expected participants with a higher risk of developing an NCD to report higher perceived efficacy and lower threat in the *coping-threat* condition (reverse order) than in the *threat-coping* condition (conventional order). We expected the opposite effect for participants with a lower risk of developing an NCD.
  2.2. Effect of experimental condition on defensive reactions
    2.2.1. We postulated that the effect of experimental condition on defensive reactions will be moderated by personal risk of developing an NCD, but we were unable to predict the direction of this effect.
  2.3. Effects of efficacy and threat on intention:
    2.3.1. We tentatively postulated that perceived efficacy would have a positive effect on intention and that perceived threat would have a negative effect on intention.
    2.3.2. We expected the effect of perceived efficacy on intention to be mediated by defensive reactions, with higher perceived efficacy leading to lower defensive reactions. However, given the conflicting results reported by previous studies, we did not try to predict the effect of defensive reactions on intention.
    2.3.3. We expected the effect of threat on intention to be mediated by defensive reactions, but we were unable to predict the direction of the effect of threat on defensive reactions or of the subsequent effect of defensive reactions on intention.

## Method

### Study design and procedure

We assessed the comparative effectiveness of three versions of the IFRC's Healthy Lifestyle Community course and investigated possible mechanisms that could account for any differences. We conducted our survey online, recruiting participants via a crowdsourcing platform. Each participant was randomly assigned to one of three versions of the course (*threat-only*, *threat-coping*, *coping-threat*). Before beginning their allotted course, participants provided demographic information and indicated any unhealthy habits they had (smoking tobacco, excess alcohol consumption, physical inactivity, unhealthy diet). Participants with no unhealthy habits were redirected to the end of the survey and thanked for taking part, because our main DV, intention to change behavior, would not have been relevant for them. Those with at least one unhealthy habit followed their allotted version of the healthy lifestyle course. Each participant was exposed to the course's generic content and to content specifically referring to that person's self-reported unhealthy habits (smoking tobacco, excess alcohol consumption, physical inactivity, unhealthy diet). They then completed a questionnaire measuring perceived efficacy, perceived threat, danger control, and fear control. The study was approved by the Ethics committee of the Faculty.

### Participants

We recruited participants via the Prolific crowdsourcing platform (Palan & Schitter, 2018). Because the IFRC's original course was intended for South American countries and written in Spanish, we recruited participants in South America. A power analysis showed that 64 subjects per condition were sufficient to detect a medium size effect of the experimental condition (dummy coded) on our main dependent variable intention, with a power of .80.^1^ In order to achieve this goal, taking into account the loss of participants, we planned to recruit 300 volunteers. Due to the fact that the platform allows new participants to begin a survey before others have finished, 377 participants started the questionnaire but only 299 of them answered all the questions. In addition, we excluded 45 participants who spent less than 5 s on pages containing text or videos^2^ and a further two participants who said they were below the age of 18. Finally, participants who did not provide data on all our study variables were excluded by PROCESS's default listwise deletion function. This gave us a final sample of *N* = 230 (66.1% men, 33.9% women) with a mean age of 27.83 years (minimum 18 years, maximum 58 years). Most of our participants (86.5%) were from Mexico; the others (13.5%) were from Chile. Repartition in the conditions was as followed: 79 in the *threat-only* condition, 67 in the *threat-coping* condition, and 84 in the *coping-threat* condition.

Table 1 presents the participants’ health habits. Very few participants had a healthy diet and a majority did less than the recommended amount of exercise. Alcohol consumption and the proportion of current smokers were quite low.

**Table 1.** Description of participants’ health habits (*N* = 230).

Smoking tobacco		Alcohol consumption				Diet		Physical inactivity	
		men		women					
Currently smoke	17.4%	5 or more units a day or more than 14 units a week	3.3%	4 units or more a day or more than 7 units a week	2.6%	Never or rarely eat fruit and vegetables	16.1%	Never exercise	17.0%
Quit	25.2%	2–4 units a day	4.6%	2–3 units a day	1.3%	Eat some fruit and vegetables, but less than 5 a day	79.6%	Exercise a little, but less than 30 min a day	46.5%
Never smoked	57.4%	1 unit a day or less than 14 units a week	92.1%	1 unit a day or less than 7 units a week	96.2%	Eat at least 5 fruit and vegetables a day	4.3%	Exercise at least 30 min a day	36.5%

### Material

The material was adapted from Units 1 and 2 of the IFRC's Healthy Lifestyle Community course. All the participants watched short videos on NCDs and risk factors. The first video (duration: 55 s) showed people with unhealthy habits and explained the consequences of these habits on their health, highlighted NCDs as the number one killer in the world, and presented the four types of NCD (cardiovascular diseases, cancer, chronic respiratory diseases, and diabetes). Participants then watched videos (one for each risk factor) explaining the health effects of each of their bad habits (smoking tobacco (1 min 30 s), drinking too much alcohol (2 min 40 s), having an unhealthy diet (1 min 46 s), being physically inactive (2 min 03 s)). Participants saw the videos about the risk factors that were relevant for them (for example, a smoker that does not drink alcohol, does eat healthy and is physically active saw only the video related to smoking tobacco).

Participants in the *threat-only* condition saw only the videos mentioned above, whereas participants in the other two conditions saw the same videos, and in addition were given coping related material: mention that the diseases were preventable, information explaining the health benefits of quitting smoking, reducing alcohol consumption, exercising, and having a healthy diet, as well as practical advices to help them change behavior, for example tips to identify serving sizes of fruits and vegetables, explanations on how to do squats, planks, and push-ups. Participants in the *threat-coping* condition (conventional order) watched the videos explaining the risks associated with unhealthy behaviors before receiving information on the health benefits of changing their behaviors and advice on how to make these changes. Participants in the *coping-threat* condition (reverse order) received the health benefits information/advice on how to change their behaviors before watching the videos explaining the risks associated with unhealthy behaviors.

### Measurements

Unless stated otherwise, responses to all items were given on five-point Likert-type scales ranging from *Totally disagree* (1) to *Totally agree* (5), plus *Don't know* (coded 99).

#### Perceived efficacy

We used four items from the scale developed by Witte (1996b) to measure response efficacy and self-efficacy, and then summed each participant's responses to give a perceived efficacy score (scores could range from 4 to 20).

*Response efficacy.* We used two items to measure response efficacy (e.g. ‘Having a heathy lifestyle can protect you from NCDs’). The reliability of the items was sub-optimal (Spearman-Brown *ρ* = .534), but because there were only two items we retained both.

*Self-efficacy.* We used two items to measure self-efficacy beliefs (e.g. ‘I am capable of having a healthy lifestyle in order to protect myself from NCDs’). Both items had satisfactory reliability (Spearman-Brown *ρ* = .775).

#### Perceived threat

We measured perceived severity of the threat and perceived susceptibility to the threat via four items adapted from Witte's scale (1996b), summing each participant's responses to give a perceived efficacy score.

*Perceived severity.* We used two items to measure perceived severity of the threat (e.g. ‘I believe that NCDs have serious negative effects’). Both items had very high reliability (Spearman-Brown *ρ* = .939).

*Perceived susceptibility.* We used two items to measure perceived susceptibility to the threat (e.g. ‘I am at risk of getting NCDs’). Both items had very high reliability (Spearman-Brown *ρ* = .858).

#### Danger control

Danger control is a cognitive process triggered by protection motivation. It involves people thinking of strategies to protect themselves from and/or avert a threat (Witte, 1996b). One possible danger control response is intention to change behavior.

*Behavioral intention.* We measured danger control via two items assessing intention to change unhealthy habits (e.g. ‘I have decided to change my habits’). We averaged scores for the two items to give an intention score (Spearman-Brown *ρ* = .802).

#### Fear control (Defensive reactions)

In the present study, we operationalized fear control, which is a coping strategy aimed at reducing the fear engendered by a threat, via the concept of defensive reactions. Although van ‘t Riet and Ruiter (2013) described four fear control strategies – avoidance, denial, suppression, reappraisal – we measured only denial, suppression, and reappraisal because it is difficult to measure avoidance in an online survey.

*Suppression.* We used two items to measure the tendency to avoid thinking about the threat (e.g. ‘When I read the information about risk factors and NCDs, my first reaction was to …  …  …  …  … think about risk factors and NCDs’). The answer scale ranged from *want to* (1) to *not want to* (5). We averaged the two items to give a suppression score (Spearman-Brown *ρ* = .788).

*Reappraisal.* We measured reappraisal via ten items from the Disengagement Beliefs Scale (Dijkstra, 2009; Dijkstra, Vries, Kok, & Rouackers, 1999). Each item is an explanation that reduces the threat, for example, ‘The risk factors may increase the chances of me developing an NCD, but I know people who had unhealthy habits and lived long lives’, and ‘The risk factors may increase the chances of me developing an NCD, but the doctors will find a cure’. This measure had good reliability (Cronbach *α* = .826).

*Denial.* We used three items to measure denial of the information given (e.g. ‘Information about risk factors and NCDs has been exaggerated’). Reliability was poor for the three items (Cronbach *α* = .501) but was improved by removing one of the items (*α* = .596), so we computed denial scores from the remaining two items.

#### Personal risk of developing NCDs

We measured each participant's risk of developing NCDs via three items, which we administered before the experimental manipulation.

*Number of unhealthy behaviors.* The first component of the personal risk measure was how many of the following unhealthy habits each participant had: smoking; drinking two or more units of alcohol a day; exercising for less than 30 min a day; and eating fewer than five fruit and vegetables a day. Each participant was attributed a score from one (i.e. one unhealthy habit) to four (i.e. four unhealthy habits).

*Family history of NCDs.* The second component was family history of NCDs, which we measured via the question: ‘Is there a history of noncommunicable diseases in your family (cardiovascular diseases, cancer, chronic respiratory diseases, diabetes)?’: No (1)/Yes (2)/Don't know/Not sure (99).

*Perceived general health.* The third component was perceived general health, which we measured via the question ‘Would you say that your health is generally: Excellent (1), Very good (2), Good (3), Regular (4), Bad (5), Do not know/Not sure (99)’.

We normalized scores for all three components so they were between 0 and 1, and then computed a personal risk score by summing the three individual scores. Hence, personal risk scores could range from 0 to 3, with higher scores indicating a higher risk of developing NCDs.

#### Ethics Statement

The study was approved by the Ethics committee of the Faculty.

## Results

### Descriptive results

Table 2 shows bivariate correlations between the possible mediators and intention to change behavior. As expected, personal risk of developing NCDs correlated negatively with perceived efficacy and positively with perceived threat, and intention to change behavior correlated positively with perceived efficacy. In addition, intention to change behavior correlated negatively with two of the fear control (defensive reaction) variables (suppression and reappraisal) and positively with the third fear control variable (denial). We obtained a similar pattern for perceived efficacy, which correlated negatively with suppression and reappraisal, and positively with denial. However, we did not obtain significant correlations between threat and any of the defensive reaction variables. Suppression correlated positively with reappraisal and negatively with denial, and denial correlated negatively with reappraisal.

**Table 2.** Descriptive statistics of the variables (*N* = 230).

	Personal risk	Intention	Efficacy	Threat	Suppression	Denial	Reappraisal
Personal risk							
Intention	−.037						
Efficacy	−.197 **	.308 ***					
Threat	.479 ***	.031	−.090				
Suppression	.022	−.524 ***	−.301 ***	−.046			
Denial	−.019	.136 *	.141 *	.005	−.166 *		
Reappraisal	.079	−.247 ***	−.192 **	.099	.264 ***	−.191 **	
	scale: 0–3 *M* = 1.47 *SD* = 0.58	scale: 1–5 *M* = 4.17 *SD* = 0.67	scale: 4–20 *M* = 17.55 *SD* = 1.95	scale: 4–20 *M* = 16.48 *SD* = 2.19	scale: 1–5 *M* = 1.54 *SD* = 0.75	scale: 1–5 *M* = 3.86 *SD* = 0.80	scale: 1–5 *M* = 3.03 *SD* = 0.70

Note: *** indicates a *p*-value < .001, ** indicates a *p*-value < .01, * indicates a *p*-value < .05.

### Hypothesis testing

We tested our mediation hypothesis via a model we created in PROCESS (Hayes, 2018) (see Syntax in appendices), version 3.3, which relies on percentile bootstrap (Biesanz, Falk, & Savalei, 2010). Results are presented in Table 3.

**Table 3.** Regression coefficients, standard errors, and summary information for the hypothesized model.

		Consequent																						
		*M_1_* Threat				*M_2_* Efficacy				*M_3_* Suppression				*M_4_* Denial				*M_5_* Reappraisal				*Y* Intention		
Antecedent		Coeff.	*SE*	*p*		Coeff.	*SE*	*p*		Coeff.	*SE*	*p*		Coeff.	*SE*	*p*		Coeff.	*SE*	*p*		Coeff.	*SE*	*p*
*X_1_* Condition Threat-only (−0.5) Threat-coping (+0.5)	*a_1_*	.11	.16	.49	*a_2_*	.11	.18	.52	*a_3_*	.03	.18	.85	*a_4_*	−.06	.17	.75	*a_5_*	−.10	.18	.57	*c’*	.18	.15	.24
*X_2_* Condition Threat-coping (−0.5) Coping-threat (+0.5)	*a_1_*	.06	.16	.70	*a_2_*	−.07	.17	.70	*a_3_*	−.12	.18	.48	*a_4_*	−.05	.17	.76	*a_5_*	−.10	.18	.58	*c’*	.07	.15	.62
*W* Personal risk	*d_1_*	**.47**	**.06**	**<.001**	*d_4_*	−**.19**	**.06**	**.004**	*d_7_*	−.01	.07	.93	*d_12_*	−.01	.07	.89	*d_17_*	.00	.08	.95	*b_1_*	.00	.06	.97
*Int_1_* Personal risk * X_1_	*d_2_*	−.14	.16	.39	*d_5_*	.20	.17	.24	*d_8_*	−.22	.18	.23	*d_13_*	−.04	.17	.82	*d_18_*	−.15	.18	.41	*b_2_*	.00	.15	.98
*Int_2_* Personal risk * X_2_	*d_3_*	−.23	.16	.15	*d_6_*	.12	.18	.49	*d_9_*	−.08	.18	.67	*d_14_*	.16	.17	.37	*d_19_*	−.09	.18	.63	*b_3_*	−.15	.15	.32
*M_1_* Perceived threat		–	–	–		–	–	–	*d_10_*	−.08	.07	.30	*d_15_*	.02	.07	.75	*d_20_*	.08	.08	.30	*b_4_*	.03	.06	.66
*M_2_* Perceived efficacy		–	–	–		–	–	–	*d_11_*	−**.32**	**.07**	**<.001**	*d_16_*	**.14**	**.07**	**.036**	*d_21_*	−**.19**	**.07**	**.008**	*b_5_*	**.15**	**.06**	**.014**
*M_3_* Suppression		–	–	–		–	–	–		–	–	–		–	–	–		–	–	–	*b_6_*	−**.42**	**.06**	**<.001**
*M_4_* Denial		–	–	–		–	–	–		–	–	–		–	–	–		–	–	–	*b_7_*	.03	.06	.63
*M_5_* Reappraisal		–	–	–		–	–	–		–	–	–		–	–	–		–	–	–	*b_8_*	−.09	.06	.11
		*R^2^* = .489 *F*(5, 224) = 14.052, *p* < .001				*R^2^* = .221 *F*(5, 224) = 2.298, *p* = .046				*R^2^* = .325 *F*(7, 222) = 3.743, *p* < .001				*R^2^* = .163 *F*(7, 222) = 0.862, *p* = .537				*R^2^* = .048 *F*(7, 222) = 1.609, *p* = .134				*R^2^* = .317 *F*(10, 219) = 10.183, *p* < .001		

Note: Effects significant at <.05 are shown in bold.

Our first result is the absence of an effect of experimental condition: neither adding coping information (*threat-coping* condition) to the *threat-only* condition, nor reversing the order of the threat and coping information (*coping-threat* condition) had an impact on any of the mediators or on intention to change behavior, our main dependent variable. Consequently, all the following effects are merely associations and causal links cannot be inferred from our data.

Perceived efficacy was significantly associated with the defensive reaction variables: higher levels of efficacy were associated with lower levels of suppression and reappraisal but, interestingly, with higher levels of denial. Efficacy was also significantly and positively linked with intention to change behavior.

Contrary to our expectations, we did not obtain significant associations between threat and any of the defensive reaction variables or intention to change behavior. In addition, the only defensive reaction variable to be significantly associated with intention to change behavior was suppression: higher levels of suppression were associated with less intention to change behavior.

**Figure 2.**
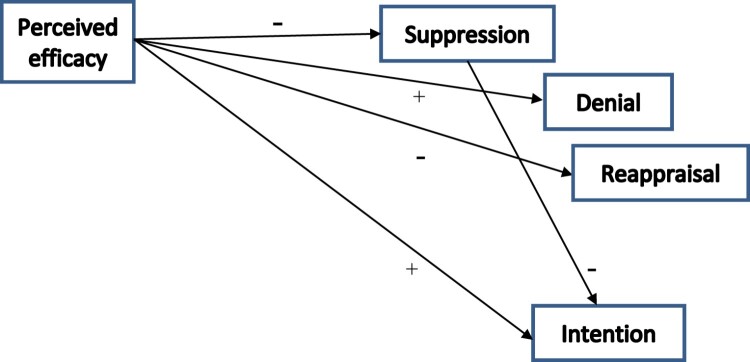
Diagram showing statistically significant relationships between the variables.

Figure 2 shows the statistically significant links in our initial model.

## Discussion

The present study compared the impacts of three different versions of an NCD prevention course on danger control and fear control reactions. Our data did not support the basic tenet that a prevention message will be more likely to prompt intentions to change behavior when it combines coping information with threat information than when it contains just threat information. Nevertheless, the fact that the *p*-value for the impact of coping information was not significant cannot be taken as indicating a total lack of effect (Dienes, 2014). In addition, our study was configured to observe a mean effect size (*f* = .25) that was larger than the effect we observed (*f* = .18). It is therefore possible that the effect of our independent variable exists but is smaller than we expected. In addition, the power analysis was done on the main effect of the experimental condition on intention, and not the full mediation model. Thus, our sample is possibly underpowered to test such a complex model. However, in order to avoid contributing to the ‘file drawer problem’, we believe it is important to publish this negative result so it can be included in any future meta-analysis. An examination of the Failsafe N (FSN) values (i.e. the number of null-findings needed to make a specific effect non-significant) reported in Milne et al.’s (2000) meta-analysis, led Conner and Norman (2005) to stress that the only robust effect was for self-efficacy, although the FSN values for coping-related variables were higher than they were for threat-related variables. Thus, despite innumerable studies of the effectiveness of fear appeals, data remain equivocal. Given the widespread use of fear appeals in health communication, it is important to note that its effects have been studied mostly via laboratory experiments (notable exceptions include Dijkstra & Bos, 2015; and Schüz, Eid, Schüz, & Ferguson, 2016), and the inconsistent nature of results suggest that such studies are not necessarily applicable to the ‘real world’.

Because our experimental manipulation did not have an effect on intention to change behavior, all remaining results are correlational. Nevertheless, we obtained an interesting pattern of results with respect to perceived efficacy and the defensive reaction variables. As expected, participants with higher perceived efficacy scores also had higher intention to change behavior scores. Perceived efficacy was also associated with lower levels of suppression and reappraisal, and higher levels of denial. Only suppression was associated with intention to change behavior: participants with higher levels of suppression had lower intention to change behavior scores. The mixed positive and negative effects of perceived efficacy on the defensive reaction variables are consistent with recent findings on the possible adaptive functions of defensive reactions. For example, van ‘t Riet and Ruiter (2013) suggested that defensive reactions may arise in parallel with coping reactions. Moreover, Blondé and Girandola’s (2016) Revised Stage Model suggests that defensive reactions may be not antithetical to effective persuasion and may even promote acceptance of health recommendations. A subsequent study by Blondé and Girandola (2019) found that greater fear led to more persuasion and to more defensive reactions (operationalized through comparative optimism). De Hoog, Stroebe, and de Wit's meta-analysis (2007) showed that threat-induced defensive processing can be compatible with greater intention to change behavior. Lewis et al. (2010) also argued that ‘empirical evidence has shown that acceptance and rejection are not mutually exclusive outcomes’ (p. 460). This is contrary to the EPPM, according to which danger control and fear control are either-or process (Maloney, Lapinski, & Witte, 2011; Witte, 1996a). Further research on the impact of defensive reactions on behavior change is needed in order to ascertain, for example, whether there are moderators that explain why defensive reactions sometimes favor and sometimes hamper behavior change.

Conventional fear appeal communications first present the threat and then provide coping information. Keller (1999) suggested that the reverse order –coping first, then threat– should be considered as an option for the ‘unconverted’, that is, for people who have not yet adopted recommendations. Her results suggested that the reverse order is better at persuading the unconverted because it provides less opportunity to discount the threatening message. Similarly, Brown and West (2015) found that the optimal order depends on the level of threat contained in the message. In their study, the conventional order worked best for a low-distress message, whereas the reverse order was more effective for a high-distress message; a finding they attributed to the effect of sequencing in reducing attentional avoidance. However, many studies, including the present study, have not obtained an order effect. For example, Hall, Bishop, and Marteau (2006) did not find any order-related difference in the persuasive impact of an anti-smoking message (assessed as intention to stop smoking), but they did obtain a primacy-effect in that participants in the conventional order condition recalled more threat information, whereas those in the reverse order condition recalled more coping information. Leventhal and Singer (1966) examined the impact of the order of threat and coping information on increasing or reducing fear produced by stimuli of different intensities. Presenting coping information after the threat (vs. before the threat) resulted in greater fear reduction in the high-fear condition but less fear reduction in the low-fear condition. However, there was no significant difference in message acceptance. Similarly, Prentice-Dunn, Floyd, and Flournoy (2001) found that varying the message order did not significantly affect behavior intentions, but participants in the conventional order, high-threat condition expressed less hopelessness than those in the high threat, reverse order condition. Finally, Skilbeck, Tulips, and Ley (1977) found that the conventional order resulted in significantly better compliance with dietary instructions at two-week follow-up, but not at four-, eight-, or sixteen-week follow-up. Hence, the current literature does not allow any firm conclusions to be drawn about which message order is most likely to induce behavior change.

An important aspect of our study is that it involved participants from low-and middle-income countries, which are understudied in the scientific literature. According to the WHO (2018), 85% of NCD-related premature deaths occur in low- and middle-income countries, so it is essential to determine whether theories and models, most of which have been developed in WEIRD (Western, Educated, Industrialized, Rich, and Democratic) societies (Henrich, Heine, & Norenzayan, 2010), are valid in lower-income countries. What is more, our study used ‘real-life’ materials, rather than materials created by researchers, which may lack ecological validity.

However, participants were paid to take to the study. Thus, their readiness to change might be different than if we recruited participants that were motivated to take part because they wanted to improve their health. Readiness to change has been mentioned in the literature as a moderator of fear appeals (Mongeau, 2013). In that sense, generalizability of our results to a motivated population is questioned. This specificity of the sample might also be another reason for the lack of effect of our experimental manipulation. However, it is worthy to mention that studies have shown that data from Prolific is of good quality: participants are more naïve and more diverse than on Amazon Mechanical Turk (Peer, Brandimarte, Samat, & Acquisti, 2017), and obviously more so than convenience sample of undergraduates students in psychology (Buhrmester, Kwang, & Gosling, 2011). A limitation of our study is that we measured behavior change via a proxy – intention to change behavior. Although intention is considered the most proximal determinant of behavior (Fishbein & Ajzen, 1975), there is a large gap between intention and behavior (Sheeran, 2002), so creating intentions is not always enough to change behavior. Our decision to measure intentions was imposed by the impossibility of measuring actual behaviors via an online survey, but it would be better for future studies to use behavior as the dependent variable. Another improvement possibility is in using more reliable scales, as low internal consistency occurred notably for the response efficacy and denial scales.

We were not able to ascertain that participants were fully attentive to the prevention material presented. This a flaw of online study, and although we controlled for the time spent on the pages with prevention material, it is possible that some participants opened other pages of their browser, or did something else during the time the videos were playing. This is a challenge even in real life behavior change program where it is impossible to guarantee participants’ responsiveness (Dane & Schneider, 1998). Future studies in more controlled design could help exclude inattentiveness as a reason for our lack of difference among experimental conditions. Finally, we did not verify that the participants were equally aware of the four risk factors. A novel health threat might be differentially influenced by fear appeal than an already known health threat (Mongeau, 2013; Quick, LaVoie, Reynolds-Tylus, Martinez-Gonzalez, & Skurka, 2018). In the case of the four risk factors considered in this study, they seem more or less be at the same level of awareness in the population in the sense that none are ‘new’ health threat like an emerging disease, although the subjective knowledge (see Nabi, Roskos-Ewoldsen, & Dillman Carpentier, 2008) has not been verified in our sample and constitutes a limitation of the study. This is also a potential moderator that could be considered in future studies targeting multiple behaviors simultaneously.

The four healthy habits promoted in the material might have differed in how they are seen as readily protecting from the threat: while quit smoking is probably directly linked in people's mind to preventing cancer, this might be less the case for eating healthier, or getting more physical activity. Thus the health communication must provide a clear conceptual match between the behavior and the negative consequences for fear appeals to have an effect. It is one of the objectives of the prevention material created by the NGO that people learn about the link between the risk factors and the four NCDs. Thus it was presented clearly to all participants in the study what the risk factors (tobacco, alcohol, sedentarity, unhealthy diet) for the NCDs are, and thus that adopting the healthy habits (quit smoking, reduce alcohol consumption, physical activity, healthy diet) are effective way to prevent getting an NCD. However it is impossible to assume that all participants adhered to the information presented. If we had measured response efficacy for each healthy habit separately, we could have relied on data to state if this is the case or not. This is a limitation of the present paper, and future studies investing multiple behavior change should consider measure DVs, and response efficacy in particular, for each behavior separately.

## Conclusion

Our results confirm the importance of high perceived efficacy in triggering intentions to change behavior. They also support the idea that defensive reactions might not always hinder change toward more healthier habits.

## Notes

1 G-power: ANCOVA, fixed effects, main effects and interactions. Effect size *f* = 0.25, alpha = .05, power = 0.8; df = 1, number of groups = 2, number of covariates = 1.

2 Measures of the time participants spent on each page showed that some participants did not watch the videos fully or stay on the page long enough to read the information. Thus, we excluded from our analyses participants who spent less than 5 s on pages containing text or videos.

## Appendices

Syntax of the model tested in PROCESS

PROCESS

y = Zintention_score/

m = Zthreat_score Zefficacy_score Zsuppression_score Zdenial_score Zreappraisal_score/

w = Zrisque_level/

x = condition_num/

mcx = 5/

xcatcode = −0.5,0,0.5,−0.5,0,0.5/

boot = 5000/

conf = 95/

bmatrix = 1,1,0,1,1,1,1,1,1,0,1,1,1,0,0,1,1,1,1,1,1/

wmatrix = 1,1,0,1,0,0,1,0,0,0,1,0,0,0,0,1,0,0,0,0,0.
